# Rapid Turn From Cirrhosis to Encephalopathy Following COVID-19 Infection: A Cautionary Tale

**DOI:** 10.7759/cureus.22089

**Published:** 2022-02-10

**Authors:** Tutul Chowdhury, Jakia Sultana, Jui Dutta, Nicole Gousy, Khondokar N Hassan

**Affiliations:** 1 Internal Medicine, One Brooklyn Health System, New York, USA; 2 Medicine, Comilla Medical College, Cumilla, BGD; 3 Medicine, American University of Antigua, New York, USA; 4 Medicine, Bangladesh Medical College and Hospital, Dhaka, BGD

**Keywords:** covid-19, atypical covid, alcohol-related liver disease, ace-2 receptor, alcohol-related cirrhosis, overt hepatic encephalopathy

## Abstract

The coronavirus disease 2019 (COVID-19) infection has most commonly led to patients presenting with pulmonary disease, including severe acute respiratory syndrome. However, in about 14-53% of patients with a newly diagnosed COVID-19 infection, the liver is the organ most drastically affected, as opposed to the lungs. In patients with preexisting liver damage, the first symptom of a COVID-19 infection may come from worsening liver failure such as hepatic encephalopathy or worsening ascites. This case report highlights this unusual presentation of a COVID-19 infection in a patient with preexisting alcoholic liver cirrhosis. We report this case to heed warning that acutely worsening liver failure may be the first presenting symptom of a superimposed COVID-19 infection on preexisting liver disease.

## Introduction

The coronavirus disease 2019 (COVID-19) infection, caused by severe acute respiratory syndrome-related coronavirus 2 (SARS-CoV-2) and leading to severe acute respiratory syndrome, has been spreading rapidly worldwide with a deleterious effect on global health. The disease was first discovered on December 19, 2019, in Wuhan, China [1], which was later declared as a public health emergency by the World Health Organization (WHO) on January 30, 2020. This viral pandemic spreads through respiratory droplets via sneezing and coughing and has an incubation period of approximately 3-5 days [2]. The most common clinical manifestations of COVID-19 are fever, cough, dyspnea, fatigue, myalgia, and gastrointestinal symptoms [3]. Apart from the respiratory involvement, it also affects multiple organ systems such as the liver, brain, etc., especially in immunocompromised patients.

Approximately 14% to 53% of patients have been reported suffering from mild to moderate degrees of liver injury due to COVID infection without preexisting liver disease. However, the patients with prior liver disease have a significantly higher hospitalization and mortality rate due to this viral disease [4]. This is particularly true for those with preexisting hepatic cirrhosis, a disease characterized by progressive necrosis, and regeneration of hepatocytes, resulting in the weakening of the immune system and alterations to the gut-liver axis of these patients [5]. Furthermore, the affliction of COVID-19 in patients with liver disease becomes more intense as the virus attaches itself to the angiotensin-converting enzyme 2 (ACE-2) receptors, in both lung and liver bile ducts [1]. This mechanism ultimately triggers liver injury with an end result of increased ammonia levels in the blood and brain resulting in hepatic encephalopathy. In our case, the patient showed symptoms of altered mental status with a history of hepatic cirrhosis and ascites. Additionally, this patient presented with radiological evidence suggesting COVID pneumonitis, which further deteriorated the patient’s condition. Abdominal distention, bilateral lower extremity edema, abnormal metabolic panel all were consistent with the patient’s diagnosis. Focusing on such pathophysiology and clinical characteristics, this review article emphasizes COVID-19-induced hepatic encephalopathy and investigates the outcomes, lab findings, and management strategies in such patients.

## Case presentation

A 51-year-old female was brought to the ER due to altered mental status for 3 days. Her past medical history was significant for hypertension and chronic alcohol abuse leading to chronic hepatic cirrhosis with ascites. She had been suffering from significant abdominal pain and swelling for a considerable period for which she was previously hospitalized. Paracentesis was done during admission along with ascitic fluid drainage at that time. No antecedent history of trauma, falls, or head injury was found.

Clinical examination exhibited a cachectic and lethargic adult female with a toxic and disoriented state. Her vital signs were hemodynamically stable during admission. There was abdominal distension consistent with her cirrhosis with visible abdominal collateral circulation and dullness in all four quadrants. However, no tenderness and organomegaly were present and no bowel sounds were appreciated. There was bilateral lower extremity edema which was more significant in the right leg than the left. Additionally, she developed a pressure ulcer on her left hip and buttock with oozing. Bilateral ischemic ulcers were also visible approximately 3×3 cm in size on the left side and 3×2 cm on the right side which were not visibly infected. Given her altered mental state, she was admitted immediately, and endotracheal intubation was performed. Thereby she was treated with some broad-spectrum antibiotics, lactulose, and rifaximin.

After admission, a metabolic panel, a complete blood count (CBC) hemogram, and cell morphology labs were also ordered (Table 1). The significant findings included a blood urea nitrogen (BUN) level of 32.9 mg/dL, a serum Na+ level of 126 mEq/L, a brain natriuretic peptide (BNP) level of 1335 pg/mL, a procalcitonin level of 5 ng/mL, and a D-dimer level of 7650 ng/mL. Her bilirubin and aspartate aminotransferase (AST) levels were also mildly elevated. A chronic microcytic hypochromic anemia with an elevated white blood cell count was additionally reported. An abnormality was also found in the blood coagulation profile (Table 2). Her arterial blood gas (ABG) analyses were performed 4 days apart which were also significant (Table 3). Her model for end-stage liver disease (MELD) score was 24 overall.

**Table 1:** Results of the patient’s metabolic panel, a complete blood count (CBC) hemogram, and cell morphology labs that were ordered after admission WBC: white blood cell count; RBC: red blood cell count; HCT: hematocrit; HGB: hemoglobin; PLT: platelet count; MCV: mean corpuscular volume; fL: femtoliter; mg/dL: milligrams per deciliter; BUN: blood urea nitrogen; AST: aspartate aminotransferase; ALT: alanine aminotransferase; CO_2_: carbon dioxide; IU/L: International units per liter; pg/dL: picogram per deciliter

Test	Value	Reference value
WBC	13.1	3.8-10.4 (× 10^3^/µL)
HGB	9.5	Male: 13.6-16.9; female: 11.9-14.8 (g/dL)
HCT	29.6	Male: 40-50%; female: 35-43%
MCV	74.2	82.5-98 (fL)
PLT	238	Male: 152-324; female: 153-361 (× 10^3^/µL)
Creatinine	0.80	0.6-1.3 mg/dL
BUN	32.9	6-20 mg/dL
Na	126	135-147 mEq/L
K	4.4	3.5-5.5 mEq/L
Cl	94	98-106 mEq/L
CO_2_	20	23-29 mEq/L
ALT	16	10-56 IU/L
AST	41	10-40 IU/L
ALP	107.6	44 to 147 IU/L
Bilirubin	2.4	<1.0 mg/dL
BNP	1335	100 pg/mL
Procalcitonin	5	<0.1 ng/mL
D-dimer	>7650	<250 ng/mL

**Table 2:** Patient’s blood coagulation profile documented over the course of her admission INR: international normalized ratio; PTT: partial thromboplastin time; CRP: C-reactive protein; sec: seconds; mg/dL: milligrams per deciliter; ng/mL: nanograms per milliliter

Test	8 days after	9 days after	10 days after	11 days after	12 days after	13 days after	On admission	Reference value
Prothrombin time	16.4	16.5	15.9	16.6	16.5	15.6	16.8	9.8-13.4 sec
INR	1.35	1.35	1.31	1.37	1.36	1.28	1.38	0.85-1.15
PTT	46.3	41.9	40.7	43.2	37.4	34	38.6	24.9-35.9 sec
CRP							13.20	0.50-1.00 mg/dL
Ferritin							251	11.10-264 ng/mL

 

**Table 3:** Arterial blood gas analysis of the patient taken over the course of her admission FiO2: fraction of inspired oxygen; mmHg: millimeters of pressure; mmol/L: millimoles per liter; mEq/L: milliequivalents per liter

	1/13/2022	1/9/2022	Reference value
Patient information			
FiO_2_	70%	100%	100%
Arterial blood gas			
pH, arterial	7.42	7.12	7.35-7.45
pCO_2_, arterial	41.8	37.7	35-45 mmHg
pO_2_, arterial	82.3	120.0	80-100 mmHg
HCO_3_, arterial	26.3	11.7	22-26 mmol/L
Base excess, arterial	2.1	15.9	-2 to +2 mmol/L
O_2_ saturation, arterial	96.0	96.4	95 to 100%
Total Co_2_, arterial	27.5	12.8	23 to 29 mEq/L

**Figure 1:**
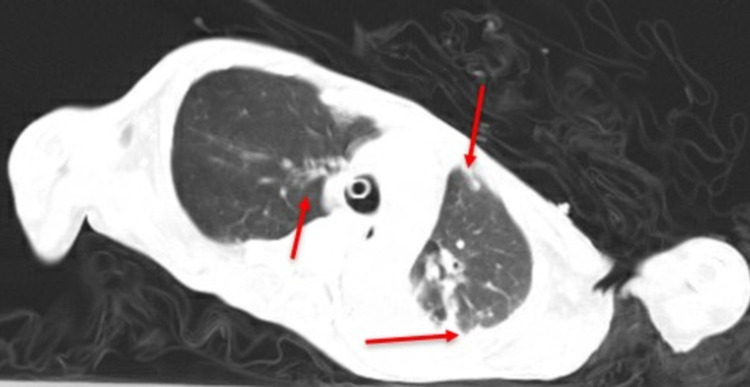
A CT scan of the chest taken during this admission showing bilateral areas of patchy airspaces and infiltrates (red arrows)

An initial chest X-ray findings showed cardiomegaly with left ventricular configuration and bilateral lower lobe infiltrate in both lobes but greatest in the left lobe. Four days later, there was extensive bilateral lung infiltration in both lobes equally. Repeated chest X-ray after 4 days showed multifocal airspace disease or pneumonia. These radiographic findings were consistent with COVID-19 pneumonitis with possible aspiration, which was confirmed later by a positive RT-PCR test. A CT scan of the chest was done to best highlight the lung infiltration (Figure 1).

**Figure 2:**
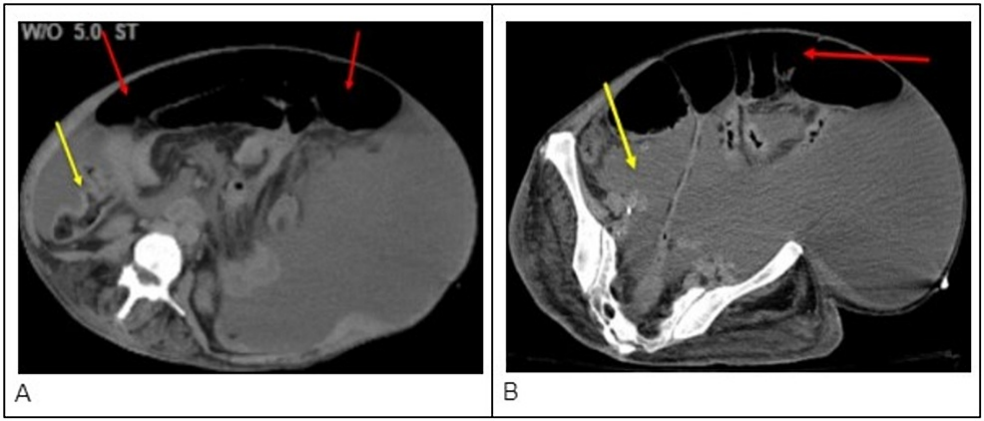
Two different CT scans of the abdomen taken during admission of this patient showing significant ascites (red arrows) and liver cirrhosis (yellow arrows)

A CT scan of the upper abdomen without contrast was also performed and showed left lower lobe atelectasis, additionally with patchy air space in the bilateral upper lobes, right middle lobe, and right lower lobe (Figure 2). Liver cirrhosis with ascites was identified. Bone erosion was found in the left inferior ischiopubic rami, suspicious for osteomyelitis. Soft-tissue swelling and soft-tissue gas were also visible in the left buttock and the left medial thigh. No well-formed abscess was identified.

Gram stain and culture of the ascitic fluid tap revealed no organism except some rare WBCs. Ascitic fluid lactate dehydrogenase (LDH) was 98, lymphocyte and macrophage levels were 28% and 9%, respectively. The urine toxicology test was positive for ethanol. According to the clinical and laboratory evaluation, a diagnosis of COVID-19 pneumonia with hepatic encephalopathy and decompensated hepatic cirrhosis due to alcohol abuse was established and therefore treated with 5% dextrose in normal saline (DNS), IV steroids, broad-spectrum antibiotics, lactulose, and rifaximin.

## Discussion

COVID-19 is caused by an enveloped, positive-sense single-stranded genomic RNA virus and spreads through close human to human contact via respiratory droplets (coughing, sneezing) [6]. In our case, the lungs were the most susceptible organ to the COVID-19 infection, followed by the liver. It has been demonstrated in several studies that lung injury by the SARS-COV2 is mediated by attaching its spike protein to the ACE2 receptor [1,6,7]. The ACE2 receptor is, incidentally, highly expressed in alveolar type-II cells, as well as liver and bile duct cells, making it possible for the SARS-COV2 to infect cells in these regions most dramatically. More specifically, cholangiocytes express the ACE2 receptor in higher concentrations than hepatocytes, making them more susceptible to viral infection [7]. However, since the liver harbors a significant amount of macrophages, thereby producing an abundant cytokine-mediated immune reaction, hepatocytes can also succumb to a SARS-CoV-2 infection, albeit concomitantly [8]. COVID-19 patients with liver cirrhosis have consistently shown elevated ALT and AST levels, elevated D-dimer levels, and elevated CRP, IL-6, and ferritin levels. In accordance with these findings, our patient also had an increased level of AST, bilirubin, CRP, and D-dimer levels.

Our case presents altered mental status in the setting of liver cirrhosis which is exacerbated after Covid-19 infection. On the onset of infection, COVID-19 can gain access to the central nervous system via the olfactory tract. Within the nasal cavity and forebrain, there is the olfactory nerve and bulb which creates an access point directly to the CNS from the nasal epithelium [9]. By utilizing this gateway, the virus can reach the brain and cerebrospinal fluid and cause inflammation and demyelination within 7 days of entering the olfactory tract through the nasal epithelium. After disruption of the blood-brain barrier by exposure to COVID-19, patients typically experience seizures, vomiting, headaches, and nausea as some of the more frequently encountered neurological symptoms [3]. In our case, there is significant hyponatremia and increased BUN which is consistent with the development of hepatic encephalopathy.

Using the MELD score, clinicians can determine the level of care for cirrhotic patients, especially those infected with COVID-19 [10,11]. While the existing literature is currently limited, studies have shown that those with chronic liver disease, such as our patient, will have elevated MELD scores and suffer increased hepatic and pulmonary complications when infected with COVID-19 [4,10,11]. Specifically, the mortality rate in patients infected with COVID-19 with preexisting alcoholic liver disease is upwards of 1.8 times higher than those without the preexisting condition. Additionally, the severity of liver damage itself due to a COVID-19 infection tends to be much more significant in those with preexisting alcoholic liver cirrhosis than those without [4]. By using the MELD score physicians can utilize risk stratification techniques to help guide treatment management more appropriately to those with elevated MELD scores. In the case of our patient with a MELD score of 24, indicating severe disease and poor prognosis, the elevated MELD score was utilized to help manage the hepatic complications, specifically the hepatic encephalopathy, that our patient presented with.

When it comes to treating patients with hepatic encephalopathy contacted by COVID-19, lactulose is the first line of treatment. After passing from the small intestine to the large intestine, lactulose is converted to lactic acid and acetic acid by bacterial action in the large gut. This creates a highly acidic environment that is unenviable to ammonia-producing bacteria. This overall decreases the amount of ammonia being created and therefore absorbed, and increases the amount of ammonium being fecally excreted. Another treatment option is the antibiotic, rifaximin, which is very effective and brings down the production of ammonia by reducing the number of ammonia-producing bacteria [11].

## Conclusions

The COVID-19 infection has been the topic of much discussion and research since it first appeared in December of 2019. While most patients infected with this virus tend to present with pulmonary complications, we present a patient who presented with severe neurological complications secondary to significant viral damage to the liver. While this is an unlikely presentation of this illness, it has been shown through numerous studies that the SARS-CoV-2 virus can infiltrate cells through the ACE2 receptor highly expressed by alveolar cells and cholangiocytes alike. In patients with preexisting liver disease, such as this patient with alcoholic liver cirrhosis, a COVID-19 infection can be detrimental to the survival of cholangiocytes and hepatocytes and propagate higher mortality rates than those without liver disease. This case highlights the significance of recognizing a COVID-19 infection as the underlying cause of severe liver damage and their unique clinical presentations.
